# Agricultural lime value chain efficiency for reducing soil acidity in Ethiopia

**DOI:** 10.1016/j.soisec.2023.100092

**Published:** 2023-06

**Authors:** Ali M. Oumer, Samuel Diro, Geremew Taye, Tadele Mamo, Moti Jaleta

**Affiliations:** a Holetta Agricultural Research Centre (HARC), Ethiopian Institute of Agricultural Research (EIAR), P.O. Box 2003, Addis Ababa, Ethiopia; b International Centre for Agricultural Research in the Dry Areas (ICARDA), Rue Hedi Karray, CP 2049, Ariana, Tunis, Tunisia; c International Maize and Wheat Improvement Centre (CIMMYT), P.O. Box 5689, Addis Ababa, Ethiopia

**Keywords:** Value chain map, SWOT, Processing plant capacity, Production cost share, Distribution channels, Lime transport

## Abstract

Soil acidity is challenging agricultural production in Ethiopia. Above 43% of the farmland is under soil acidity problem and it leads to low crop yields and production losses. Ag-lime is widely considered as an effective remedy for amending soil acidity. This study assesses the current structure of ag-lime value chain and its functionality focusing on central parts of Ethiopia where lime is produced and channeled to acidity affected areas. The study uses Ethiopia as a case study and applies qualitative methods such as key informant interviews and focus group discussions to collect data from different actors in the ag-lime value chain. Key findings indicate that both public and private ag-lime producing factories are operating below their capacity. Due to limited enabling environments, the engagement of private sector in ag-lime value chain is minimal. In addition, farmers have a good awareness of soil acidity problem on their farms, and its causes and mitigation strategies in all regions. However, the adoption of ag-lime by smallholders was minimal. Overall, the current structure of the ag-lime value chain appears fragmented and needs improvement. Addressing soil acidity challenge through efficient ag-lime value chain could narrow lime supply-demand mismatches and increase widespread adoption by farmers to enhance crop productivity and food security in acidity-prone areas of the country.

## Highlights

• Soil acidity is challenging crop production in Ethiopia.
• Ag-lime is an effective remedy for amending soil acidity globally.
• Ag-lime producing factories in Ethiopia are operating below their capacity.
• Farmers are aware of soil acidity, but lime use is minimal due to weak lime value chain.
• The structure of ag-lime value chain in Ethiopia is fragmented and needs improvement.

## Introduction

Agricultural transformation in Ethiopia requires the appropriate use of soil resources. About 43% of Ethiopia's agricultural land is affected by soil acidity and 28% is estimated to be strongly acidic with soil pH ranging between 4.1 to 5.5 (ATA, 2014; Abebe, 2007; Agegnehu et al., 2019; 2021). Soil acidity can lead to low crop yields and in severe cases, may result in complete production loss. Soil acidity has now been a well-recognized problem to increase agricultural productivity and achieve food security in large parts of the country. Abebe (2007) has reported that soil acidity is expanding in scope and magnitude across different regions of Ethiopia, particularly in the western parts of the country. Some studies have reported the severity of soil acidity, agricultural productivity challenges and ongoing initiatives to mitigate the problem in the country (e.g., Warner et al., 2016; 2023; Tamene et al., 2017; Amede et al., 2019; Agegnehu et al., 2019; 2021).

Some technologies and practices have been recommended to reclaim soil acidity and upgrade the productivity of strongly acidic soils. These include the cultivation of acid-tolerant plants, covering the surface with non-acidic soil, the use of organic fertilizers, and liming. Among these practices, liming and the application of organic fertilizers appear the best measures, because of their more persistent agronomic effects (Chen et al., 2001; Desalegn et al., 2019; Warner et al., 2023). Ag-lime is a soil conditioner made from crushed limestone or dolomitic limestone with the primary active component of calcium carbonate. Ag-lime increases the pH of acidic soil that reduces soil acidity and increases alkalinity. Such soil conditions provide calcium for plants, improve water penetration for acidic soils, and increase the uptake of major plant nutrients (nitrogen, phosphorus, and potassium).

The application of agricultural lime (ag-lime hereafter) is one of the main strategies for reducing soil acidity and increasing crop productivity. Consequently, Ethiopia has initiated an acid soil reclamation strategy that can address different segments along the ag-lime value chain (e.g., Warner et al., 2016; Amede et al., 2019). The goal of the initiative is to provide enough ag-lime to farmers at affordable prices in a timely and sustainable way. These initiatives include the production of ag-lime in the upstream segment, transportation of lime including the last-mile distribution to farms in the mid-stream segment and the use of lime by farmers in the downstream segment. Several efforts have also been made to develop acid soil management packages, increase awareness through on-farm demonstrations and adoption of liming practices (Amede et al., 2019). Despite these efforts, however, the actual ag-lime application is negligible in the country. Previous studies (e.g., Warner et al., 2016) showed that only 6% of planned agricultural lands received lime treatment, and only 7% of farmers used lime on their land in the first Growth and Transformation Plan (GTP I) period. The story was not different in GTP II. Only 36% of the planned cultivated lands were treated with ag-lime in the GTP II period (MOA, 2020). In both GTP I and GTP II, only 2.4% of the severe acid soil area (3.7 million hectares) have been treated with lime. With this trend, it may take over 200 years to fully treat the strong acidic soil of Ethiopia. Overall, efforts to treat soil acidity so far have lagged expectations and now require effective interventions.

Improving the efficiency of the ag-lime value chain requires the active participation of both public and private sector actors. However, there is limited information on the current structure of the ag-lime value chain in Ethiopia. The production, transportation and distribution of lime are not well coordinated in most cases. Therefore, understanding the underlying technological and institutional hurdles surrounding the ag-lime value chain would help provide adequate ag-lime to farmers in time and at a reasonable price. It would also create investment opportunities for the private sector actors and job creation for the public sector along the value chain to spur economic growth in the country.

The objective of this study is to analyze the current production, distribution, and utilization of ag-lime in major administrative regions of Ethiopia. The specific objective is to explore the main actors, their roles, and functions as well as opportunities and challenges along the ag-lime value chain for the development of an efficient value chain. Together with other few studies (Agegnehu et al., 2019; 2021; Warner et al., 2023), the study contributes to the development of acid soil reclamation strategy in Ethiopia that envision to bring impact like the Brazilian acid soil reclamation initiative in the Cerrado region (Yamada, 2005). Given soil acidity is a challenge to other African countries (AGRA, 2022), the study provides valuable information on the current value chain structure for enhanced food production by addressing the economics of soil health for improving farmers’ livelihoods in Ethiopia and other African countries.

The rest of the paper is organized as follows: the next sections present the methodology followed and different qualitative methods used to collect and analyze data. The third section provides a summary of major findings and discussion. The paper concludes with some reflections and policy implications.

## Methodology

### The study areas

The study was conducted in four major crop-dominated regional states of the country. These were the Oromia, Amhara, Southern Nations, Nationalities and Peoples (SNNP), and Sidama regional states. These regional states were selected because of two major reasons. First, the state-owned/public ag-lime factories are established in these regions except for the Sidama regional state. These public lime factories are producing, supplying ag-lime, and promoting related improved agricultural practices and knowledge to farmers. Second, there is high coverage of soil acidity in these regions. Small and large-scale lime demonstrations have also been conducted in these regions and encouraging ag-lime use practices have been reported.

### Research design

This study uses Ethiopia as a case study to provide useful information about ag-lime production, distribution, and utilization for acid soil management in the smallholder farming systems. The government of Ethiopia has established ag-lime factories in Oromia, Amhara, and SNNP regional states to increase ag-lime production and supply to enhance crop productivity in areas affected with severe soil acidity. The study also focused on three state-owned (public) lime crushing factories: Guder, Dejen, and Kella lime factories in Oromia, Amhara, and SNNP regional states, respectively. These factories were established to reclaim acidic soil zones in the country. In addition, we assessed a few private lime processing factories (Dashen and Muger cement factories) which have started producing ag-lime. Important information and data were obtained from the Bureau of Agriculture (BoA) and farmers utilizing ag-lime technology in the West Shoa, East Gojjam, and Sidama zones. We also assessed the status of ag-lime awareness and utilization in the study areas. Insights from this ag-lime value chain analysis contribute to the ongoing broader efforts in guiding acid soil management investments in Africa (AGRA, 2022; Agegnehu et al., 2021; Warner et al., 2023).

### Data, data collection methods, and analysis

We reviewed literature and reports related to ag-lime use in Ethiopia. Qualitative research methods were used to collect data from major actors and facilitators of the ag-lime value chain. We used a purposive sampling procedure to select the main stakeholders/actors involved in the ag-lime value chain for data collection (Table 1). The main actors included the Ministry of Agriculture (MoA), the respective regional, zonal, and district level Bureaus of Agriculture (BoA), smallholder farmers, public and private ag-lime crushers, Non-Governmental Organizations (NGOs), the Ministry of Mines (MoM), input supplying cooperative unions, and research centers found in the regional states. Data were collected from these actors using semi-structured checklists for key informant interviews (KII) and focus group discussion (FGD). The FGD was used to assess farmers’ perception of soil acidity and ag-lime use in the study regions. Strengths, weaknesses, opportunities, and threats (SWOT) analysis was conducted to understand the public and private ag-lime production and ag-lime distribution process in detail. A few zonal and woreda level BoAs were purposely sampled to examine the distribution of ag-lime at the grassroots level. Visual observations of the factory sites and a few phone interviews were also done to refine critical information.

**Table 1:** Number of respondents interviewed in each of the lime value chain actors.

Stakeholders/Actors		Number of respondents interviewed at government administrative level					
		Federal	Oromia Region	Amhara Region	SNNP Region	Sidama Region	Total
Ministry of Mines (MoM)		2					2
Ministry of Agriculture (MoA)		3					3
Regional Bureau of Agriculture (BoA)			3		2	2	7
Zonal BoA			3	3			6
Woreda BoA			3	3		2	8
Cooperative Unions			3	3		2	8
Non-governmental organization (NGO)			3				3
Private lime crushers (primarily cement factories)			2	2			4
State-owned lime crushers (ag-lime producers)			5	2	3		10
Research Institutes		2					2
Farmers	Key informant interviews (KII)		3	9			12
	Focus group discussions (FGDs)		8	9		7	24

The qualitative data were recorded in handwritten notes and immediately transcribed or typed into the computer for further content and thematic analysis. The qualitative data collected from various actors in the ag-lime value chain was categorized and organized using a spreadsheet. The information was then organized within each research question according to a predetermined theme or category. This helped to document relevant information and spot issues that are frequently raised by the various actors in the value chain. Text information was also thoroughly analyzed using both content and thematic analysis to pinpoint certain themes and emergent patterns. We narrated the information following the themes created to generate the report. Finally, we identified the voices of the various actors in the value chain to enrich the dynamics, validity, and reliability of findings (Aveling et al., 2015).

We estimated costs and margins of ag-lime business along the production and distribution nodes of the value chain starting from core-limestone suppliers to distributors. The cost information was gathered from major actors including core-limestone suppliers, ag-lime factories, transporters, and distributors. Furthermore, we assessed farmers’ cost of production at the Gozamen district in the East Gojjam zone using key informant interviews. We also analyzed the effect of ag-lime adoption on the yield and revenue of farmers in the selected district. The average wheat yield (productivity) was sourced from Gozamen district BoA. Data related to wheat productivity of land treated with lime was collected from the key informant farmers in the district. Wheat was considered for the analysis because of its dominance in the study areas.

We summarized the main results using figures, tables, and charts. Ag-lime production, distribution and utilization trends were also shown. Descriptive statistics were used to summarize the ag-lime cost of production and gross margins along the value chain. We present the major findings and discussions in subsequent sections.

## Results and discussion

### Farmers’ perception of soil acidity

According to farmers, soil acidity and fertility challenges were previously treated using local soil management practices such as fallowing cropland to improve the productivity of their land. However, the practice of fallowing cropland has limited prospect because of land scarcity and unsustainability to address the problem of soil acidity in the long-term (Warner et al., 2023).

Our qualitative results indicate that farmers are aware of soil acidity problems and mitigation strategies. Farmers learn about soil acidity from development agents (DA) and researchers. Tailored training on soil acidity was also provided to some farmers and then passed to fellow farmers. Farmers can also detect soil acidity on their land through poor crop growth, and low productivity even if inorganic fertilizer is applied. Complete crop failures due to soil acidity are also observed by farmers. Farmers perceive soil erosion and over-plowing (continuous cropping without leaving crop residues) as the main causes of soil acidity. Different soil acidity mitigation strategies are also used by farmers as reflected during the focus group discussion. These include the use of compost and manure, crop rotation with legumes and ag-lime application. Farmers produce wild oats locally known as *'sinar'* and plant eucalyptus trees on non-responsive farmlands because of severe soil acidity. These crops are considered tolerant to soil acidity. Farmers are also aware that the severity of soil acidity affects even the growing period of eucalyptus trees. Concerning this, a key informant farmer from the Oromia region said:

*"The severity of soil acidity is increasing from time to time in our area. Our land is becoming out of production, and we are changing annual crops into eucalyptus trees. Even the harvesting time of eucalyptus trees are affected by the problem. Moderately acidic soil takes four years and highly acidic soil takes six years for harvesting even the eucalyptus trees planted on these fields”.*

During the FGD, farmers at Amhara region indicated low use of lime technology due to different reasons. Amongst, high price of ag-lime and cost of application were the major ones. They added, initially, government and non-government organizations introduced the ag-lime technology free of charge for popularization purpose. However, currently farmers have stated that they are paying huge amounts of money to get lime needed for their land. They confirmed that there is access to a credit service for ag-lime, despite the high interest rate. The lack of small innovative farm implements to apply lime is also a constraint for the adoption of lime technologies, added the farmers.

A key informant farmer from the Amhara region also shared the points raised during the FGD. He said,

*“Transportation from the distribution center to home and to farmland costs us money. This additional cost of ag-lime purchase, interest rate, transportation, and application cost inflates our cost of production and reduces our profit margin since the price of grain we sell is not increasing accordingly. Due to this, some farmers used to plant eucalyptus trees on their land which is crop and land damaging harmful tree. This could create conflict between neighborhood farmers who have adjacent farmlands”.*

Regarding the intensity of soil acidity, the key informant farmers perceive that 60–75% of land in their village is affected by soil acidity. Yet, few farmers (10–15%) use ag-lime to reclaim their land in their respective *kebeles*.^1^ The focus group discussants indicated that farmers in the study areas apply ag-lime during the sowing period in early-July in Oromia region, late-June to early-July in Amhara region, and mid-June to early-July in Sidama region.

Farmers in the Oromia region sow barley, wheat, and teff on lime-treated lands. Farmers in the Sidama region sow barley, wheat, potato, and beans. The choice of these crops depends on their importance to food security or cash as well as advice by DAs. Farmers perceive that ag-lime alone could not increase much crop yield unless it is complemented by other inputs such as improved seed, fertilizers, and proper land preparation. This farmers’ perception is consistent with recent studies recommending targeted lime application as part of a greater policy of integrated soil fertility management that includes fertilizer and farm management strategies (Agegnehu et al., 2019; 2021; Gurmessa, 2021; Warner et al., 2023).

The key informant farmers noted ag-lime adoption barrier in the study areas. These include the bulky nature of lime, the difficulty of transportation to farm plots (lack of simple machines), labor-intensive nature of the lime application, untimely (late) supply of lime, and low supply to remote *kebeles*. Farmers do not apply ag-lime on rented-in land because of uncertainty to use the rented-in land for subsequent seasons when the soil acidity is reduced. Farmers suggest timely supply of lime, access to spreading machines, credit, and subsidies for a widespread use of ag-lime. These support services have also been noted and suggested by different studies (Warner et al., 2016; Tamene et al., 2017; Gurmessa, 2021; AGRA, 2022).

### Ag-lime production in Ethiopia

Ag-lime is produced by both private- and public-owned lime processing plants. However, both the public and private ag-lime processing factories are operating below their potential capacity (Table 2). Government-owned lime crushers (Guder, Dejen and Kella lime factories) were supposed to operate at full capacity. However, due to limited demand and other internal operational challenges, the three factories are operating at sub-optimal capacity. Dashen cement factory has a large ag-lime processing capacity but during 2021, it produced only 2% of its maximum capacity. The factory produced only the quantity of lime contracted to it by the Amhara Regional Government for the 2020/2021 production season. Likewise, Muger cement factory has the largest processing capacity but produced less than 1% of its maximum capacity due to a lack of ag-lime demand. Given the existing potentials in ag-lime processing at the factories established mainly for lime crushing and the cement factories that could easily adjust and produce lime for agriculture, we (the authors) argue that producing a large quantity of ag-lime should not be an issue in Ethiopia.

**Table 2:** Overview and utilized capacity of lime processing plants visited in Ethiopia.

Factory name	Ownership	Location (Administrative Zone)	Capacity (tons/year)	Production in 2021 (tons)	Production relative to capacity (%)
Guder lime factory	Public (Oromia BoA)	West Shoa	46,800	5687	12
Dejen lime factory	Public (Amhara BoA)	East Gojjam	7200	1400	19
Dashen lime factory	Private	East Gojjam	144,000	3000	2
Kella lime factory	Public (SNNP BoA)	Guraghe	5414	1585	29
Muger cement factory	Public (Chemical Industry corporation)	West Shoa	400,000	2500	1

Source: field visit to lime factories, 2021.

Lack of autonomy on financial management and key decisions by the government-owned lime crushers were reported by the public lime factory managers as a major cause for low amount of production even when there is some level of demand. Guder, Dejen, and Kella lime factories were established by the government of Ethiopia to support ag-lime supply for the acid soil treatment initiatives. According to the lime factory managers, these factories get their operational budget from the respective District Finance Offices they are in, and any income generated from the lime sale also goes to the same offices. Any procurement decisions (including spare parts for machinery maintenance) are passed through the government offices. Managers of these factories indicated the inefficiency created especially where there is any maintenance problem during the peak lime processing seasons (e.g., Guder and Dejen factories). We also observe that storage problems for produced ag-lime and electric power interruptions to be critical in all the public lime factories.

The private lime factory managers indicated that private lime processors need a guaranteed product market and yet are not ready to take any risk in lime marketing. As the market for ag-lime is not yet well developed, the private lime processors are reserved from taking any marketing risks and stick to the contract agreement they have with the regional governments for the quantity of agricultural lime they produce. We noted that these private factories are not taking any costs in promoting and marketing ag-lime. They just respond to the regional governments’ procurement plan and produce what is agreed upon between them. That could be one of the critical reasons for the disparity between their maximum annual production capacity and their actual production in a season.

Table 3 presents the SWOT analysis of public ag-lime factories. The SWOT results indicate that lack of warehouses, power interruption and lengthy financial processes are the major weaknesses in state-owned ag-lime production factories. If the production node of the value chain can be improved, we observe that these factories can increase lime production to their maximum capacity. However, access to abundant labor, core-limestone deposit, government, and NGOs attention to soil acidity could be seen as opportunity for sustainable ag-lime production in future.

**Table 3:** SWOT analysis of public ag-lime producing factories.

**Strengths**	**Weaknesses**
• Availability of better crushing machines and capital-intensive features of the factories.	• Lack of stores (warehouses) for produced lime. • Packaging materials are expensive. • Power interruptions are common. • Lack of essential experts such as mechanic and chemists. • Poor financial system (poor purchase process). • No transportation trucks for ag-lime. • No standard for ag-lime quality control (only calcium carbonate equivalence done).

**Opportunities**	**Threats**
• NGOs' current involvement in enhancing ag-lime production. • Plenty of labor supply in production areas. • Huge core-limestone deposits in all production areas. • Big attention by the government for ag-lime value chain development. • Growing lime production experiences.	• Security (political instability). • Lime supply-demand mismatch.

Source: field visit to lime factories, 2021.

Table 4 presents the SWOT analysis of private ag-lime factories. The SWOT results indicate that there is an increasing cost of production due to inflation in prices of inputs such as packaging material, fuel and lubricants, and labor that put threats to the private ag-lime factories. Surprisingly, our SWOT analysis indicate that the private ag-lime factories have much capacity to produce but there is low effective demand for the ag-lime. We noted that this could be a result of poor coordination and governance in the ag-lime value chain. Creating an ag-lime stakeholders’ platform and coordination could help address the ag-lime production supply-demand mismatches as also noted by previous studies (Warner et al., 2016; Tamene et al., 2017; AGRA, 2022). We argue that the stakeholders’ platform can also address the problem of information asymmetry about ag-lime demand for suppliers (ag-lime crushers and distributors) to make informed production/stocking decisions and reduce the carry-overs. In this regard, the Brazilian acid-soil reclamation initiative was able to bring together diverse stakeholders such as the government, the national research institute, and international technical and financial aid to carry out an intensive rehabilitation initiative that resulted in the reclamation of over 60 million hectares of farmland and created a global production source for wheat (Warner et al., 2023).

**Table 4:** SWOT analysis of private ag-lime producing factories.

**Strengths**	**Weaknesses**
• Huge capacity and capability to produce ag-lime. • Have the potential to provide transportation services. • Huge storage capacity in the factory.	• Electric power interruptions. • Low demand of ag-lime (quantity requested annually is very low). • Lack of support from government and NGOs.

**Opportunities** • Huge core limestone deposits in all production areas.	**Threats** • The high cost of production could halt farmers' demand.

Source: field visit to lime factories, 2021.

Overall, our results indicate that the lime crushing plants we visited are producing much less than their potential capacity because of limited demand, lack of storage capacity, power supply shortage, etc. Our key findings corroborate with the view that effective policies are required to incorporate effective incentive mechanisms (Gurmessa, 2021) for increasing the supply of ag-lime to their maximum capacities.

### Ag-lime technology popularization

The regional and zonal level agricultural extension experts we interviewed indicate that several extension methods are used to popularize ag-lime technologies in Ethiopia. This is expected to create demand for ag-lime by farmers. For example, the MoA Soil Resources Development Desk provides training of trainers (ToT) to regional states once a year on the management of acid soils. Furthermore, the Directorate uses public media for transmitting tailored programs regarding acid soil management and the ag-lime technology. In all regions, the major popularization methods were large-and small-scale demonstrations, field days, public media, leaflets, cluster farming and training on soil acidity and its management strategies including liming as reflected by the key informant agricultural extension experts.

We noted that these ag-lime technology popularization efforts are done by a range of stakeholders including research institutes, Bureaus of Agriculture (BoAs), and NGOs particularly, the Germen Technical Cooperation (GIZ). The Ethiopian Institute of Agricultural Research (EIAR) also promotes ag-lime through small-scale and large-scale demonstrations, field-days, training, and cluster-farming. Different electronic media are also used for the extension of ag-lime technologies to farmers. NGOs such as GIZ also support the demonstration of lime technologies to farmers and popularize ag-lime through field days, mass media, farmers' field schools, posters, and pamphlets. Different radio and TV programs, news and documentaries have been transmitted regarding the use of ag-lime in collaboration with the BoA. Despite these efforts, lack of demand is the major limiting factor contributing to the low adoption of ag-lime in the country, said the experts. In addition, for smallholder farmers, lime is a new and unusual production input that was not common before some years. Farmers were struggling to even use the commonly known production inputs such as improved seeds and fertilizers. Unlike fertilizer and seed, the volume of ag-lime needed per unit of area is quite huge which makes the purchase and transportation difficult. Due to such reasons, convincing farmers to purchase ag-lime is a difficult process, added the experts.

An expert at GIZ working on acid soil management remarked that farmers’ dependency is the major constraint towards the low adoption of lime technology in the country. Research and development partners popularize any technology for free on fields of few farmers to supplement effective extension. Then, government extension takes the lead to scale out the technology for the masses. Farmers must be aware of this as there is ‘no free lunch’, added the expert.

Mass popularization is not the case for all regions. Unlike in the Amhara and Oromia regions where lime technology is widely popularized, lack of lime popularization was raised as the main constraint in the Sidama region. For instance, the manager of Elto Union stated that the Union faced 40% carryover which was due to low demand for ag-lime. They were ordered by BoA to supply ag-lime but the demand was low due to limited popularization and mobilization done in the Sidama zone by the relevant stakeholders. The carryover increases the cost of storage and could further inflate the price of lime. In addition, access to credit was a problem in the region as stated by the manager.

We stress that consolidated efforts in ag-lime popularization are also vital in future through the integration of key actors in the value chain to enhance this sluggish demand. This is because the benefits from the investments of lime are not always clear (or immediate) to resource-poor farmers as it takes few years for the benefits to accrue, and yet the awareness campaigns and extension services have not been able to materialize this notion. For example, Warner et al. (2023) find that fertilizers cost over two times as much as a single application of lime over a five-year period for wheat in highly acidic soils of Ethiopia. These cost savings on the use of lime reaches as high as 121% of the average one-year agricultural household income for wheat. However, these long-term ag-lime benefits are not apparent for smallholder farmers to motivate their adoption. In Kenya, information and knowledge gap were created on when and how to apply lime because of mixed messages shared with farmers from various organizations that promote lime use (KMT, 2020). Thus, we underline that lime related extension and popularization efforts should be coordinated to maximize awareness and widespread adoption by farmers.

### Ag-lime transportation and distribution

**Fig. 1:**
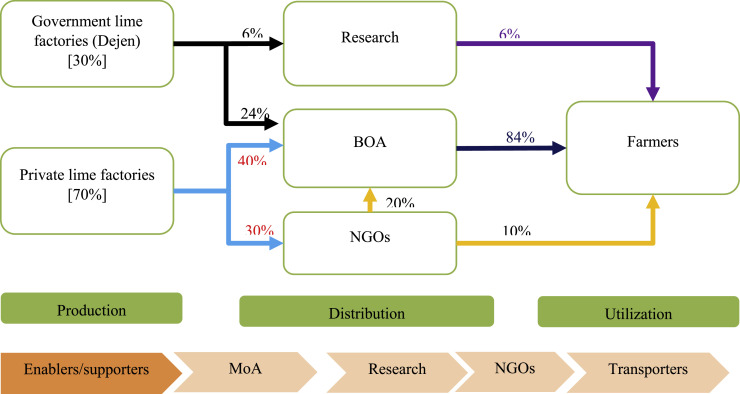
Ag-lime value chain map in the Amhara region. The figure shows the ag-lime transportation and distribution channels in the Amhara region.

Lime transportation and distribution are critical at the mid-stream node of the value chain. Different supply chain modalities were found in the regional states covered under this study. In the Amhara Region, the main lime supplier is the private lime factory (70%) and the public supply accounts for 30% (Fig. 1). Though there is some limited support from the regional government in transporting lime from lime factories to the nearby lime stores (cooperative unions), farmers are paying part of the actual price of lime. The BoA accounts for 84% of the lime distribution to farmers. The contribution of NGOs and research institutes in the distribution process is minimal accounting for 10% and 6%, respectively.

**Fig. 2:**
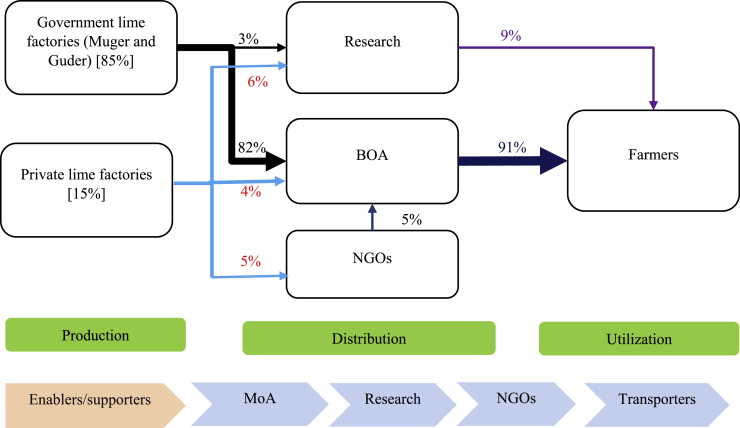
Ag-lime value chain map in the Oromia region. The figure shows the ag-lime transportation and distribution channels in the Oromia region.

However, in Oromia Region, the regional government is fully subsidizing the production and distribution of lime where farmers get lime free of charge through the district BoA. The public sector supplies 85% of the lime while the private sector accounts for only 15% (Fig. 2). The BoA distributes 91% of the ag-lime to farmers while research institutes account for only 9%.^2^

We observe that free handing of lime could help for promotional purposes and awareness creation among farmers operating on acid soils. However, prolonged free handing could discourage lime business development by deterring the involvement of the private sector. It appears that the current ag-lime value chain is quite traditional. We find that the ag-lime value chain in all public crushing sites is highly fragmented with short actor chains. This result was consistent with Warner et al. (2016) who also found fragmented and uncoordinated ag-lime value chains in major acid soil areas of Ethiopia. There should be actionable interventions transforming the current ag-lime value chain toward a more coordinated and integrated business venture.

**Fig. 3:**
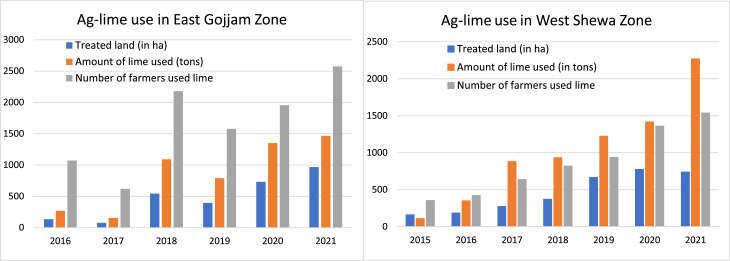
Ag-lime use in East Gojam and West Shewa Zones of Amhara and Oromia regions, respectively.

Nevertheless, there is a gradual increase in the number of farmers applying lime on their farms, the quantity of lime used, and land area treated by lime each year in the major study areas (Fig. 3). The major study areas are in the East Gojjam and West Shoa zones where the public-owned Dejen and Guder lime factories are located. Most of the products from these factories are used for acid soil management in their regions and beyond. Though these numbers are a small proportion of the number of farmers and lands affected by soil acidity in these zones, the observed progress in lime use appears encouraging.

According to the government owned ag-lime factory managers, the poor distribution system has also challenged the production volume of ag-lime in Ethiopia. Distribution is ordered from the Regional Bureau of Agriculture and quota based for zones and districts. They should go to Addis Ababa and Bahirdar (for Oromia and Amhara Regions, respectively) to come with a letter of permission to purchase the lime, the managers added. This long process for distribution discourages the demand and increases the time of storage in the factory's warehouses. For instance, the manager of Guder ag-lime factory from Oromia region stated that timely distribution is one of the major problems to expand ag-lime production. He explained the problem as follows.

“*The production activity of ag-lime in our factory is throughout the year. However, demand is limited to a few months ranging from April to June. Thus, our warehouse is filled with the product, and we store it in open areas outside the warehouses as lime is bulky and needs a huge space for storage. Failing to distribute on time caused lime produced to be damaged by rain. We stopped some machines from working to reduce the production volume due to a lack of demand, low capacity of warehouses, and untimely distribution*”.

Table 5 presents the summary of the SWOT analysis for ag-lime distributors. The ag-lime distributors included extension, NGOs, and research. Based on the discussion with key informants of ag-lime distributors, we identified key strengths, weaknesses, available opportunities, and threats in the lime distribution node that need policy action.

**Table 5:** SWOT analysis of ag-lime distributors.

**Strengths**	**Weaknesses**
• Growing demand for ag-lime. • Better linkage with NGOs. • Better awareness by farmers. • Cluster-based farming popularization. • Trials ongoing to change bulk to blend. • Involvement of private factories in ag-lime production. • Better government attention and commitment to acid soil management.	• Budget shortage for extension, demonstrations, and laboratory services. • Low laboratory capacity and facilities. • Delayed laboratory results for soil samples testing. • Lack of ag-lime spreading tools and machines. • High demand-supply mismatch due to poor planning. • Untimely supply of ag-lime to woredas and farmers. • High ag-lime transportation cost. • Lack of credit services for ag-lime. • No ag-lime minimum quality standard in Ethiopia.

**Opportunities** • NGOs attention to popularizing ag-lime. • Contribution of private ag-lime producers (cement factories and others). • Research support in knowledge transfer. • Better farmers’ awareness of soil acidity. • Involvement of cooperative unions in transportation and distribution. • High government attention to soil acidity and liming.	**Threats** • Demand-supply mismatch can lead to crop failure. • Ag-lime cost of production could inflate its price. • Bulky nature of ag-lime push transportation cost. • Increase in soil acidity intensity: moderate acidic areas are becoming highly acidic. • Carryover effects of ag-lime by regional BoA could cause dumping and quality deterioration. • High transport costs inflate ag-lime purchase costs.

Source: Discussion with ag-lime distributors (extension, research, and NGOs), 2021.

In summary, we emphasize the importance of exploiting the strengths/opportunities and addressing the weaknesses in the ag-lime current distribution process to narrow the supply-demand disparities. Encouraging the private sector (e.g., agro-dealers, private lime crushers, etc.) through targeted incentives could create trust gradually and sustain the ag-lime distribution and marketing system. In the future, we expect that granular forms of ag-lime (KMT, 2020) could also be sought to make the transportation and distribution process much easier to meet farmers’ ag-lime demand.

### Value chain enablers and facilitators

We observed that several organizations and institutions support the ag-lime value chain in the country. These organizations empower and facilitate the value chain so that farmers would have an adequate and sustainable supply of lime at a reasonable price. The main actors include the national ag-lime technology extension by the Ministry of Agriculture (MoA) and BoAs, research institutions, NGOs, cooperative unions, and the Ministry of Mines (MoM). We briefly describe the main actors and their current roles.

The national ag-lime technology extension is mainly done by the Ministry of Agriculture (MoA) and the BoAs in the major regional states. The MoA also prepares and distributes different manuals, pamphlets, and leaflets for regions which help build the capacity of experts and DAs. Continuous follow-ups, monitoring, and evaluations of implemented demonstration activities were also the major activities of the ministry as reflected by the key informant agricultural experts. The ministry also uses stakeholder platforms for ag-lime distribution and dissemination. The BoAs in Oromia, Amhara, SNNP and Sidama regions also undertake the ag-lime extension in their respective zones, woredas and *kebeles* despite differences in implementing capacities. In all regions, we learnt from the key informant agricultural extension experts that the management of acid soils was given high priority in the extension system, and they have allocated soil fertility experts in their respective zones and woredas. Soil acidity reclamation has been included in the annual training programs for farmers, DAs, and experts. NGOs such as GIZ and the Sustainable Land Management (SLM) program support these trainings. The DAs and model farmers also deliver the training to fellow farmers in their respective *kebeles*. According to the key informant agricultural experts, special emphasis was given to acid soil management in different soil fertility management training because of its significant effect on crop production and productivity to enhance the food security of the country.

We also noted that research institutions contribute to the development of the ag-lime value chain. The main contributions include the generation and popularization of ag-lime technologies, building the capacity of experts, DAs, and farmers, and soil acidity testing services.^3^ The EIAR provided a range of intensive training for various stakeholders every year in major acid-prone areas (Table 6). Currently, EIAR is introducing and testing ag-lime spreading machines that can be attached to tractors and save farm labor in lime application.

**Table 6:** Training provided by EIAR centers on acid soil management from 2019 to 2021.

Participants		2019	2020	2021
Number of regions		4	4	5
Number of zones		9	9	9
Number of woredas		13	19	28
Number of farmers	Male	1014	364	3028
	Female	258	229	638
Number of experts and DAs		288	188	418

Source: National Acid Soil Research of EIAR, 2021.

NGOs support the ag-lime value chain in major acid-soil prone regions of the country. They support the BoAs through training, providing pH meters, supplying ag-lime, ag-lime crushing machines and other physical capacity building efforts. The main NGOs and projects working on acid soil management efforts include GIZ, the Alliance for a Green Revolution in Africa (AGRA), and Agricultural Growth Program (AGP) supported by the 10.13039/100004421World Bank.

We noted that the cooperatives and unions are just beginning to engage in the ag-lime value chain. In general, the cooperative and unions provide little marketing and supply of ag-lime to farmers. Nonetheless, we observed the significant role of cooperatives and unions in the transportation and distribution of ag-lime. These include *Robi Berga* Union in Oromia, *Elto* Farmers’ Union in Sidama and *Gozamen* Farmers’ Union in the Amhara region. We observed that these Unions and associated primary cooperatives had experience in the distribution of inorganic fertilizers and could also handle the distribution of ag-lime. However, ag-lime demand should be created for farmers and cash liquidity constraints are addressed for well-functioning of the distribution process. With its current bulky nature, an average of 20 quintals (Qt)^4^ of lime is required to treat a hectare of land affected by soil acidity. Thus, transportation and distribution would be a daunting task for the actors involved in the process and could require targeted incentives or subsidies as also argued in a recent study (AGRA, 2022).

Ministry of Mines (MoM) provides licenses for mining industries based on different criteria and documents. Several mining companies produce non-ag-lime but are now interested in producing ag-lime if there is a substantial demand as noted by key informants from the MoM (Table A.1). For instance, Homa Construction PLC, and Derba Lime and Chemical PLC showed a great interest in producing ag-lime if there is effective demand from the government in large quantities and quality. Therefore, we note that these mining companies offer good opportunities for ag-lime supply in the country. We argue that engaging them in the ag-lime value chain would be crucial through a well-coordinated stakeholders’ platform.

### Soil laboratory services

Different soil laboratories at national and regional levels provide soil sample testing for woredas and *kebeles*, and lime quality testing services for lime producing industries. National soil laboratory of Ethiopia usually tests the quality of lime produced at the ag-lime-producing factories. Soil laboratory services are provided by the national soil laboratory, regional soil laboratories and research centers’ soil laboratories. Calcium carbonate (CaCO_3_) equivalence is the major chemical attribute observed by the laboratory tests. The national soil laboratory sometimes suggests that the factories should not use deposits of certain locations if calcium carbonate equivalence is low at that specific location.

Several public-owned regional soil laboratories serve woredas and *kebeles* in testing the acidity level of the soil sample for free. The samples were sent to the laboratories through the woredas, and the results would be sent back to the woredas immediately. Delayed laboratory results were the major challenge raised by woredas during this assessment. In addition, research centers of EIAR provide soil test services for woredas and *kebeles*. For instance, Holetta Agricultural Research Center (HARC) provides soil test services for 2–4 zones and 2–5 woredas annually. In 2018/2019, the center tested 320 soil samples for different acid soil prone zones and woredas. Frequently, service receiving zones include West Shoa, North Shoa (Oromia), and Finfinnee Special zone. Due to the limited capacity of soil laboratories and increasing demand for soil test results, delays of soil test results were encountered in most of the study areas according to a key informant from the research institute. Additional soil laboratories, at least in each of the zones, are important in addition to capacitating the existing soil laboratories. Soil testing is crucial for a targeted application of ag-lime and to avoid blanket use that has huge cost implications.

### Costs and gross margins along the value chain

We present the costs and margins across each actor along the ag-lime supply chain. The major actors include core-limestone suppliers, ag-lime factories, transporters, and distributors. Most of the ag-lime factories supply core-limestone by themselves except the Kella ag-lime factory. The estimates of the cost of core-limestone supply is based on the information gathered from the Kella lime factory. The private core-limestone supplier won the bid to supply one cubic meter (m^3^) of limestone for 47 ETB or 5.5 ETB/Qt of limestone. According to the suppliers, 40% of the gross income goes to costs such as fuel, machines, and renting trucks.

Ag-lime production factories are the key actors in the lime value chain. The government ag-lime factories have different cost structures (Table 7 and Table 8) and options for improved efficiency could vary.^5^ For the Dejen lime factory, the wage for casual staff is the major cost component (55%). It is followed by packaging and labeling (16%) and maintenance and repair (7%). The cost-share for the Kella factory is quite different from the Dejen lime factory. The major share of costs at the Kella lime factory are wages to contract staff (41%) followed by packaging and labeling (20%) and salary to permanent staff (15%). Kella lime factory is a capital-intensive factory compared to the Dejen lime factory. Our analyses indicate that it could be possible to cut significant costs by mechanizing the Dejen lime factory while recruiting skilled permanent staff for the Kella lime factory. Furthermore, bulk transport could reduce packing and labeling costs given there are shades to store powder lime supplied in bulk. These cost reductions ultimately translate to lower lime prices that farmers pay.

**Table 7:** Costs of ag-lime production at Dejen lime factory.

Cost items	Annual cost (ETB)	Cost-share (%)
Wages to causal staff	1371,753	54.6
Packaging and labeling	392,000	15.5
Maintenance and repair	178,000	7.1
Fuel and lubricants	150,000	6
Salary to permanent staff	111,648	4.4
Wages to contract staff	89,712	3.6
Electricity	80,000	3.2
Miscellaneous costs	141,000	5.6
Total	**2514,113**	**100.0**

Source: Dejen ag-lime factory, 2022.

**Table 8:** Costs of ag-lime production at Kella factory.

Cost items	Annual cost (ETB)	Cost-share (%)
Wages to contract staff	1286,688	41.4
Packaging and labeling	634,000	20.4
Salary to permanent staff	459,300	14.8
Maintenance and repair	230,000	7.4
Fuel and lubricants	150,000	4.8
Supply of core limestone	87,175	2.8
Uniform, clothing, and bedding	258,600	8.4
Total	**3105,763**	**100.0**

Source: Kella ag-lime factory, 2021.

The costs of transporters include the truck rental cost, fuel, and unloading cost. In Oromia, SNNP, and Sidama regions, the average transport cost of ag-lime to *Kebeles* is 163 ETB/Qt. The average margin of transporters in these regions was 35% and the unloading cost was 10 ETB/Qt. Thus, the net margin is 57 ETB/Qt for transporters in these regions. In the east Gojam zone of the Amhara region, the average cost of transportation to *kebeles* was 60 ETB/Qt. According to the key informant transporters in the region, the net transportation cost (payment for truck and fuel) is 23 ETB/Qt, and the unloading cost was 10 ETB/Qt. The rest, i.e., 27 ETB/Qt is the net margin of the transporters. Thus, the average margin of transporters in all regions was 42 ETB/Qt. The cost of transportation is calculated based on distances in kilometers ag-lime is moved. In the Oromia region, the transportation cost was 0.55–0.75 ETB/Qt/km and Elto Union serves at 0.30 ETB/Qt/km for the *kebeles* in the Sidama region. We used the average transportation costs to *kebeles* at 42 ETB/Qt for the gross margin calculation.

Primary and secondary distributors operate in the ag-lime supply chain. The primary distributors are mainly BoAs and unions. They obtain lime from ag-lime factories or NGOs and distribute it to primary cooperatives (final distributors). However, BoAs sometimes distribute by themselves in some regions such as in the Oromia region. Moreover, they purchase lime for 200 ETB/Qt in Oromia, spend 10 ETB/Qt for unloading and get a margin of 20 ETB/Qt. In the Amhara region, the unions purchase at 280 ETB/Qt and sell at 300 ETB/Qt with a margin of 20 ETB/Qt. The transport cost, loading and unloading costs are covered by transporters.

The final distributors are mainly primary cooperatives that receive ag-lime from primary distributors such as BoAs and/or cooperative unions. They get the lime for 300 ETB/Qt and sell it to farmers at 320 ETB/Qt. The difference of 20 ETB/Qt is the commission or margin they collect for the distribution of lime. This implies that the final distributors have no cost incurred to ag-lime storage.

#### Costs of ag-lime users (farmers)

We present the costs of ag-lime for wheat production in the study area (Table 9). Wheat was a crop considered for the study because of its dominance in the study *kebele*. On average, it costs 46,230 ETB to produce wheat on a hectare of land. The cost of ag-lime purchase, transportation from cooperatives to home, ag-lime application, and mixing costs are new costs due to soil acidity which is estimated to be 2860 ETB. The average quantity of ag-lime applied for a hectare of land in the area is 20 Qt. A split application of ag-lime is used in the region for four years.^6^ A split application of ag-lime could lessen the cost burden of resource-poor farmers who could not afford to pay at once if they were to apply the full rate at once.

**Table 9:** Cost of wheat production with ag-lime in Gozamen district of East Gojjam zone.

Costs per hectare	Unit	Quantity	Unit cost	Total cost
Ag-lime	Quintal	5	320	1600
Lime transport from coops to home	Quintal	5	12	60
Labor for lime application	Person/day	2	200	400
Oxen for mixing lime	Number of pair oxen	4	200	800
Seed	Quintal	2.6	3600	9360
NPS	Quintal	4	1600	6400
UREA	Quintal	2	1590	3180
Herbicide	Liters	1	300	300
Farm labor	Person/day	92	200	18,400
Farm oxen (Opportunity cost)	Number of pair oxen	20	200	4000
Transportation	Quintal produced	31	30	930
Marketing cost	Quintal sold	20	40	800
Total cost				46,230

Source: Gozamen district farmers – key informant interview (KII) result, 2021.

**Fig. 4:**
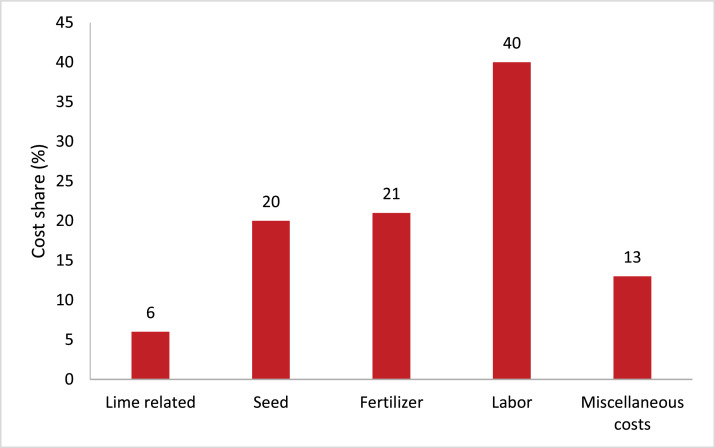
Share of input costs for wheat production in Gozamen district of East Gojjam zone. Source: FGD result with key informant farmers, 2021.

The largest share of farmers' wheat production costs goes to labor (40%) followed by fertilizer (21%) and seed (20%). With split ag-lime application method, lime-related costs are estimated to be 6% which is a new cost for farmers because of the increasing soil acidity (Fig. 4). However, if the farmers were to use the full rate of lime at once, the cost related to lime would have risen to above 30% (i.e., 5 times 6%). Therefore, the split application of lime in the study area can help reduce the cost burden to farmers.

#### Gross margin of production and distribution segments

A total of 138.35 ETB/Qt gross margin was gained from core-limestone suppliers to final ag-lime distributors. The largest share of gross margin goes to ag-lime factories which was 36.7% (50.8 ETB/Qt) followed by transporters (31.9% or 44.25 ETB/Qt). Both primary and final distributors of ag-lime gain a margin of 20 ETB/Qt or 14.5% of the total gross margin generated in the value chain. Core-limestone suppliers gain 2.4% of the gross margin (3.3 ETB/Qt) (Table 10). The processing and transporting of lime take the major share of the profit margins made along the value chain. Therefore, our analyses imply that any intervention in improving efficiency in ag-lime production and distribution could help reduce lime prices paid by farmers. These nodes of the supply chain are where large capital costs are involved and increasing efficiency through economies of scale could help reduce these costs.

**Table 10:** Gross margin analysis of lime along the value chain (ETB/Qt).

Value chain stage	Total cost	Selling price (charging)	Gross margin	Share of the final price paid by farmers (%)
Core-limestone supply	2.2	5.5	3.3	2.4
Lime Factory	159.2	210.0	50.8	36.7
Transport	93.3	137.5	44.3	31.9
Primary distributors	280.0	300.0	20.0	14.5
Final distributors	300.0	320.0	20.0	14.5

Source: Computation based on data collected from field visit, 2021.

#### Farmers’ ag-lime cost and gross margin

Farmers in the study area predominantly grow wheat on lime-treated plots. Accordingly, the yield of wheat for ag-lime user farmers was 12.5% more than the wheat yield of non-users. Our finding is consistent with result of Holland and Behrendt (2020) who found a positive impact of lime treatment on wheat yield in the United Kingdom. Hijbeek et al. (2021) also found that liming significantly improved maize yield in Kenya. Likewise, the cost of ag-lime users was 6.6% more than non-users. The net revenue of ag-lime users is 17.5% more than that of non-users. The result implies that though the use of ag-lime inflates the cost of production, the net revenue farmers’ gain in the end is higher than the increased cost (Table 11).

**Table 11:** Effect of use of lime on cost and revenue of farmers.

Particulars	Yield (Qt/ha)	Price of wheat (January 2022) (ETB/Qt)	Gross revenue (ETB/ha)	Cost for lime users (ETB/ha)	Cost if lime is not used (ETB/ha)	Net revenue (ETB/ha)
East Gojjam average	32	2950	94,400		43,370	51,030
Lime users at Gozamen district	36	2950	106,200	46,230		59,970

Source: Key informant interview, Gozamen district BoA and researchers’ computation, 2022.

## Conclusions and policy implications

The objective of this study was to analyze the ag-lime value chain in Ethiopia around three major lime factory sites: Guder, Dejen, and Kella. We collected data from different stakeholders to examine how the current ag-lime value chain is structured, integrated, and functioned. Ag-lime is produced both by public and privately owned factories in the country. However, BoAs are not well handling the public lime factories to the expected levels. Consequently, there is underproduction of ag-lime and not utilizing what is produced due to poor planning and distribution process. These problems appear to be managed in the private lime factories although these still need support to exploit their maximum production capacity. Muger cement factory and private Dashen ag-lime factory have huge potential to produce required quantities due to their ample core-limestone deposit. However, these factories are also producing below 3% of their capacity due to low ag-lime demand. Thus, increasing the production capacity of public lime factories would be crucial. Engaging private cement factories in the ag-lime production could also significantly enhance widespread lime supply in the country.

The structure of the value chains around the three public factory sites is quite different. The ag-lime value chain in Amhara appears well developed compared to the other regions. Likewise, the adoption of ag-lime is better in Amhara region than in other regions. This is because farmers in the Amhara region have started to buy lime through credit or cash depending on their capacities. Conversely, farmers in other regions are still freely getting limited quantities of ag-lime.

Government extension, research, NGOs, universities, and cooperatives are among the key enabling institutions involved in the popularization and distribution of ag-lime. These institutions offer training, build physical capacity, conduct soil acidity tests, and provide free lime for demonstrations. Cooperative unions especially in Amhara and Sidama regions also support the ag-lime value chain in transporting and distributing ag-lime to users. However, there is no strong integration among the actors and often each actor attempts to address the problem alone. There is no joint planning and implementation to address the soil acidity problems in a well-coordinated and integrated manner. Therefore, improving the interaction, linkages, and networking among main actors in the value chain would be crucial. This requires creating and managing a well-coordinated ag-lime stakeholders’ platform to address the supply and demand challenges simultaneously.

Ag-lime technology demonstrations and farmers’ advisory services are still needed. In some areas, farmers' awareness about the benefit of lime is quite low due to limited popularization work done so far. This calls for intensive awareness creation both for local agricultural experts and farmers through a tailored approach. It is also better to involve farmers in participatory ag-lime demonstration and field experience sharing. The participation of farmers in field days and on-farm demonstrations would create more awareness on the benefits of lime and enhance widespread ag-lime utilization in acid-soil prone areas.

Farmers in the Amhara region reported that lime is expensive, and they are getting it only through purchase in cash. In this regard, it is important to actively involve and capacitate cooperative unions in the lime value chain through credit arrangements. It would also be important to build cooperatives’ capacity to enhance the lime transportation and distribution to farmers. Currently, the role of cooperatives in the ag-lime value chain is very limited, especially in Oromia and SNNP regions. Ag-lime distribution should be started like fertilizers at the *kebele* level for acid-soil prone areas. In the future, granular forms of lime could help reduce the distribution and application hurdles.

Last-mile lime distribution is another challenge farmers are facing in lime use. Lime is bulky and competes with agricultural labor during peak periods such as planting. It also burdens women and children who mainly participate in the last-mile transportation and application of lime on farm plots. In reducing drudgery in the application of lime, it is also essential to improve farmers’ access to small farm implements and machinery that can save labor.

Strong planning is required in the ag-lime value chain. Public ag-lime factories are challenged by a shortage of stores and limited warehouse capacity. BoAs often distribute ag-lime for farmers close to the planting season (usually May) and negatively affecting crop production. Thus, timely distribution of ag-lime is crucial. The current planning process is quite weak.

Farmers should be encouraged to use ag-lime in combination with inorganic fertilizers and soil-water conservation practices such as manure and compost application, and the retention of crop residues. It should be noted that the application of ag-lime alone would not increase much production unless complemented with fertilizers and soil-water conservation measures. This is because once the lime is applied to the field, its residual effect could stay longer period especially if the plot is not exposed to erosion. We recommend policymakers to pursue a bundle of strategies promoting ag-lime and related technologies as also argued by other studies (Agegnehu et al., 2019; 2021; Gurmessa, 2021; Warner et al., 2023) through stakeholders’ innovation platforms to boost agricultural productivity and ensure food security in Ethiopia and beyond.

## Declaration of Competing Interest

The authors declare that they have no known competing financial interests or personal relationships that could have appeared to influence the work reported in this paper.

## Data Availability

Data will be made available on request.

## Appendix Supplementary materials

Image, application 1

## Footnotes

1 *Kebele* is the lowest administrative unit next to district (*woreda*) in Ethiopia.

2 We assessed the lime supply modalities in SNNP and Sidama Regional States. Similarly, free distribution of ag-lime through BoA is practiced.

3 Ag-lime technologies and popularization by EIAR can be found in Desalegen et al. (2019).

4 One quintal equals 100 kilograms.

5 The cost data from the Guder ag-lime factory was a lump sum and hence we did not include it in the calculations.

6 It should be noted that once the full recommended rate of lime is applied, it can benefit farmers for up to five years. In this region, farmers use a split application, and the costs and yield benefits are calculated for a single year. The total cost for lime would have been five times the single year if they were to opt for the full rate of lime at once.
