# Supplementary material for: Agricultural lime value chain efficiency for reducing soil acidity in Ethiopia
Source: Soil Secur. 2023 Jun;11:None. doi: 10.1016/j.soisec.2023.100092 (PMC10332288; doi:10.1016/j.soisec.2023.100092)
Supplement: Supplementary file 1 [file mmc1.docx]

**Appendix**

Table A.1. Registered mining companies that could produce ag-lime in Ethiopia

| No. | Name of Company | License type | Location of production |
| --- | --- | --- | --- |
| 1 | Modern building industries PLC | Small scale | Harari |
| 2 | East Cement Share company | Large scale | Oromia, North Shoa, Hidabu Abote |
| 3 | Derba Midroc Cement PLC | Large scale | Oromia, West Shoa, Ada Berga |
| 4 | Abay Industry Development Share Company | Large scale | Amhara, East Gojam, Dejen |
| 5 | Inchini Bedrock Cement | Large scale | Oromia, West Shoa, Ada Berga |
| 6 | Derba Lime and Chemicals PLC | Large scale | Oromia, West Shoa, Ada Berga |
| 7 | Habesha Cement Share Company | Large scale | Oromia, West Shoa, Meta Robi |
| 8 | Homa Construction PLC | Large scale | Oromia, West Shoa, Ada Berga |
| 9 | Dangote Industries PLC | Large scale | Oromia, West Shoa, Ada Berga |
| 10 | Mengsteab Industrial and Commercial PLC | Large scale | Oromia, North Shoa, Kuyu |
| 11 | Pioneer Cement Manufacturing PLC | Large scale | Dire Dawa |

Source: Ministry of Mines, 2021
